# The Digital Mind: New Concepts in Mental Health 1: The promise of a model-based psychiatry: building computational models of mental ill health

**DOI:** 10.1016/S2589-7500(22)00152-2

**Published:** 2022-10-10

**Authors:** Tobias U Hauser, Vasilisa Skvortsova, Munmun De Choudhury, Nikolaos Koutsouleris

**Affiliations:** Max Planck UCL Centre for Computational Psychiatry and Ageing Research, University College London, London, UK (T U Hauser PhD, V Skvortsova PhD); Department of Psychiatry and Psychotherapy, Tübingen Center for Mental Health (TüCMH), Medical School and University Hospital, Eberhard Karls University of Tübingen, Tübingen, Germany (T U Hauser); Wellcome Centre for Human Neuroimaging, University College London, London, UK (T U Hauser, V Skvortsova); School of Interactive Computing, Georgia Institute of Technology, Atlanta GA, USA (M D Choudhury PhD); Section for Precision Psychiatry, Department of Psychiatry and Psychotherapy, Ludwig-Maximilian-University, Munich, Germany (N Koutsouleris MD); Institute of Psychiatry, Psychology and Neuroscience, King’s College London, London, UK (N Koutsouleris); Max Planck Institute of Psychiatry, Munich, Germany (Prof N Koutsouleris)

## Abstract

Computational models have great potential to revolutionise psychiatry research and clinical practice. These models are now used across multiple subfields, including computational psychiatry and precision psychiatry. Their goals vary from understanding mechanisms underlying disorders to deriving reliable classification and personalised predictions. Rapid growth of new tools and data sources (eg, digital data, gamification, and social media) requires an understanding of the constraints and advantages of different modelling approaches in psychiatry. In this Series paper, we take a critical look at the range of computational models that are used in psychiatry and evaluate their advantages and disadvantages for different purposes and data sources. We describe mechanism-driven and mechanism-agnostic computational models and discuss how interpretability of models is crucial for clinical translation. Based on these evaluations, we provide recommendations on how to build computational models that are clinically useful.

## Introduction

Over the past decade, computational models have become more prominent in psychiatric research and—aligned with the fourth industrial revolution—are also finding their way into clinical and commercial solutions for psychiatry. In this Series paper, we chart the landscape of computational models in psychiatry, highlight the communalities and differences between different types of computational models, discuss their advantages and disadvantages for research and clinical practice, and distinguish between mechanism-driven and mechanism-agnostic models, which have traditionally served different purposes. Mechanism-driven models are biology-inspired models that mimic processes in the brain and are interpretable in their mechanisms. Conversely, mechanism-agnostic models use complex machine-learning methods to distil information from large datasets and often provide little insight into the relevant mechanisms. Here we show that these model types are complementary and describe how models from both domains can be brought together to build more interpretable models that are more likely to find a place in clinical practice than using each model-type in isolation.

## The digital psychiatrist

The COVID-19 pandemic has inadvertently put mental health into the spotlight. Psychiatric symptoms have strongly increased and the demand for remedies is higher than ever.^1,2^ These changes have not gone unnoticed in the corporate sector. Mental health solutions are more popular than ever and startups in mental health have become a hot commodity. Companies that pursue automated and online-based solutions have gained much attraction from investors, and technology giants, such as Apple, have ventured into predicting mental health problems using our ever-present smartphones.^3^

At the core of this excitement is the promise that computational approaches can help improve and broaden access to mental illness detection, prediction, and intervention. However, computational approaches to psychiatry are already well established in academic research, with the fields of computational psychiatry (panel 1) and precision psychiatry existing for almost a decade.^4^ In the first paper in this Series, we will selectively review the different computational approaches and their respective data sources that have been used in academic research. Rather than present a systematic literature review, we will provide a narrative description of the field and illustrate what we consider important contributions using selected examples from computational psychiatry and precision psychiatry. Although a delineation of these two fields is not clear cut and the terms are sometimes used interchangeably, traditionally computational psychiatry has focused more on understanding the mechanisms underlying mental disorders whereas precision psychiatry has focused on prediction and individualised treatment. We discuss how different modelling approaches can be meaningfully brought together to overcome limitations and move towards clinically useful models. As academics, clinicians, and the industry are moving closer together, computational approaches could be greatly beneficial, but an in-depth crosstalk between these different fields is essential to build meaningful models.

## What are the application areas of computational modelling in psychiatry?

Computational modelling in psychiatry aims to achieve different objectives that can be roughly divided into four categories.^4–7^

### Mechanism

Many academic studies aim to understand the biological mechanisms that cause mental illness, often investigating the neural mechanisms that underpin mental disorders. The goal of these approaches is to understand how processes in the brain go wrong, which can facilitate the development of better biomarkers for diagnosis, prevention, and therapeutic intervention.^4,5,7^

### Subtyping

A longstanding challenge for psychiatry is that we know little about the biological causes of mental health problems. Current diagnostic manuals are not informed by any neurobiological mechanisms, and their purely descriptive analyses of symptoms have been criticised because of doubts of the validity of diagnostic labels.^8^ Therefore, there is hope that computational models will be able to deconvolve the heterogeneity of psychiatric disease taxonomy by generating new measures that are more objective and biologically driven.^4,5^ These approaches largely rely on unsupervised models, such as clustering, aimed at discovering meaningful patterns in the data that are then evaluated against external measures, like treatment outcomes.

### Status prediction

An important goal is to predict a mental health status, either concurrently or before the development of disease to predict the changes that are about to emerge.^9^ Predicting mental illness before its development is particularly important because it might allow the prevention of adverse disease courses in a timely and efficient manner. These endeavours are most commonly used in the early psychosis field, in which high-risk states are well established, providing highly valuable windows of opportunity for preventive interventions.^10^

### Treatment stratification

From a therapeutic perspective, predicting which patient will benefit from a particular treatment is essential. Psychiatry has developed a variety of non-pharmacological and pharmacological treatments, but a substantial proportion of patients will not benefit from these treatments. Finding out which patients benefit from a specific treatment is often a tedious and slow trial and error process. Therefore, the hope is that computational models can help improve treatment predictions, be it either to select between different types of therapeutic strategies (eg, psychotherapy *vs* medication) or to select the specific form of treatment (eg, selective serotonin reuptake inhibitors *vs* serotonin and noradrenaline reuptake inhibitors).

## Computational models: from mechanism-agnostic to mechanism-driven models

### Why do we need computational models?

Computational models attempt to structure information using mathematical equations. By doing so, computational models describe a lawful association between a set of input variables (eg, neural activity, self-reported outcomes, and smartphone geolocations [panel 2]) and one or multiple output variables (eg, behaviour, psychiatric diagnosis, and treatment response). Because these associations are specified mathematically, computational models can quantify how well they capture these output variables (ie, model fit), and even simulate such outputs, which allows us to interrogate these systems in silico to better understand how they work.

The elegance of computational models is primarily in their ability to detect meaningful hidden patterns in complex data. Often, mental health-relevant information is not directly observable in collected raw data (eg, brain activity or current social media usage), but only through aggregating this input data can one extract clinically useful patterns (eg, information processing biases in the brain and stereotyped behaviours). Therefore, the function of computational models is to condense and aggregate data, but also to determine the structure of meaningful variation, which can help forecast clinically relevant developments.

**Figure 1:**
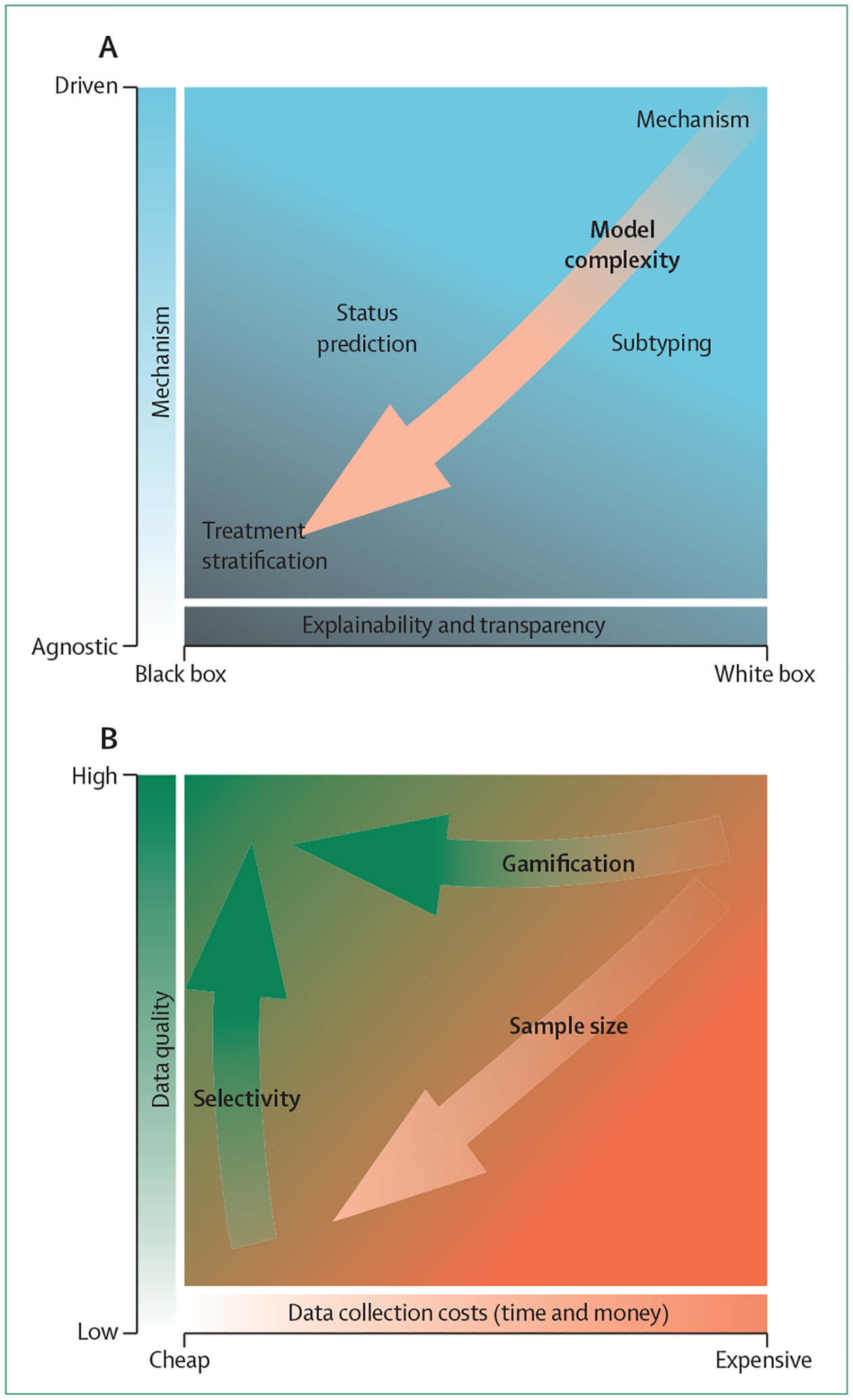
Trade-offs between models and data sources (A) Models differ in their transparency of the mechanisms, which determines their best use. Although most complex models often achieve higher predictive performance, white box models allow an understanding of the underlying mechanisms. (B) The choice of data source matters. High quality data (such as laboratory experimental studies) are often expensive (eg, functional MRI). Passive data collection is inexpensive, but the features are often unclear and not well defined. By transforming laboratory-based methods (eg, using gamification), substantially larger datasets can be collected at lower costs.

In this Series paper, we sort computational models according to how they are mechanistically formulated (figure 1A). On one hand, mechanism-agnostic models provide no information about how input variables meaningfully relate to or explain output variables—in machine learning these models are termed black box models because the model creator is oblivious about how the model works.^20^ On the other hand are mechanism-driven models, also known as white box or glass box models,^21^ for which the link between input and output variables is clearly described and directly observable from the model formulation.

### Mechanism-driven computational models

A key goal of academic research in mental health is to understand why psychiatric disorders arise and what the neural underpinnings and mechanisms are. To this end, researchers combine neuroscience methods (eg, functional MRI) with computational modelling. These models are inspired by our knowledge about the brain function and imitate the information processing that takes place in the brain.

Due to brain complexity, most computational neuro-scientists do not attempt to replicate the brain one to one, but use abstractions based on principles that are known to guide brain function. This allows the computational models to remain interpretable. A key challenge for this approach in modelling mental ill health is to determine the right level of abstraction. If a psychiatric disorder arises from an ion channel impairment, then these channels should be explicitly characterised in the model. However, if a breakdown takes place at the level of communication between different hierarchically organised brain regions, then modelling single synapses and neurons is probably not necessary and they can be approximated as entire ensembles.^22,23^ Thus far, computational psychiatry has seen approaches at many different levels of abstraction,^23–27^ but a superiority for one level of abstraction has yet to be shown.

**Figure 2:**
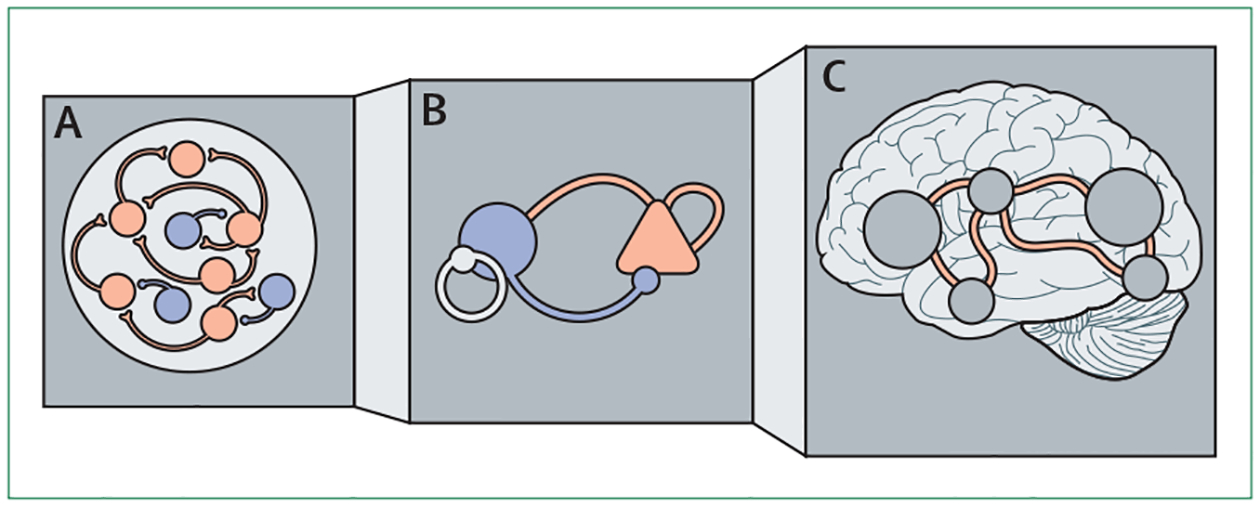
Mechanistic models of brain function Schematic representation of different levels of abstraction used in modelling brain functioning from spiking network models (A) to neural populations (B) to models incorporating multiple brain regions (C).

Some of the most exciting recent insights are from approaches that allow movement between different levels of abstraction, allowing models to map processes spanning different layers of disease pathology. Spiking neural networks with hundreds of neurons can be simplified while keeping many of the key features and the versatility of the original models.^24–28^ Such models of neuronal populations can then be used to go beyond single brain regions and model the interactions between regions and even whole brain connectivity (figure 2).^28,29^ Having translatable models at these different levels of abstraction is also appealing because they can accommodate distinct brain recording modalities.

These network models are of great promise because they can capture key features of psychiatric disorders (such as schizophrenia),^30–33^ and extensions even allow modelling specific neurotransmitters directly. One can now assess how specific drugs can affect brain functioning and work towards finding the best possible treatment on the basis of a patient’s specific network imbalances.^34–36^ These models provide a mechanistic insight into brain function and dysfunction, but might also be useful for informing psychiatry about new biologically driven subtypes and help to predict treatment response.

A second set of mechanism-driven modelling approaches focuses on capturing behaviour as closely as possible and is less tightly connected to the specific brain implementation. Specifically, reinforcement learning, Bayesian, and similar models are promising for representing complex behaviours and behavioural biases in patients and linking behaviour with subjective experiences and clinically relevant symptoms.^37–40^

**Figure 3:**
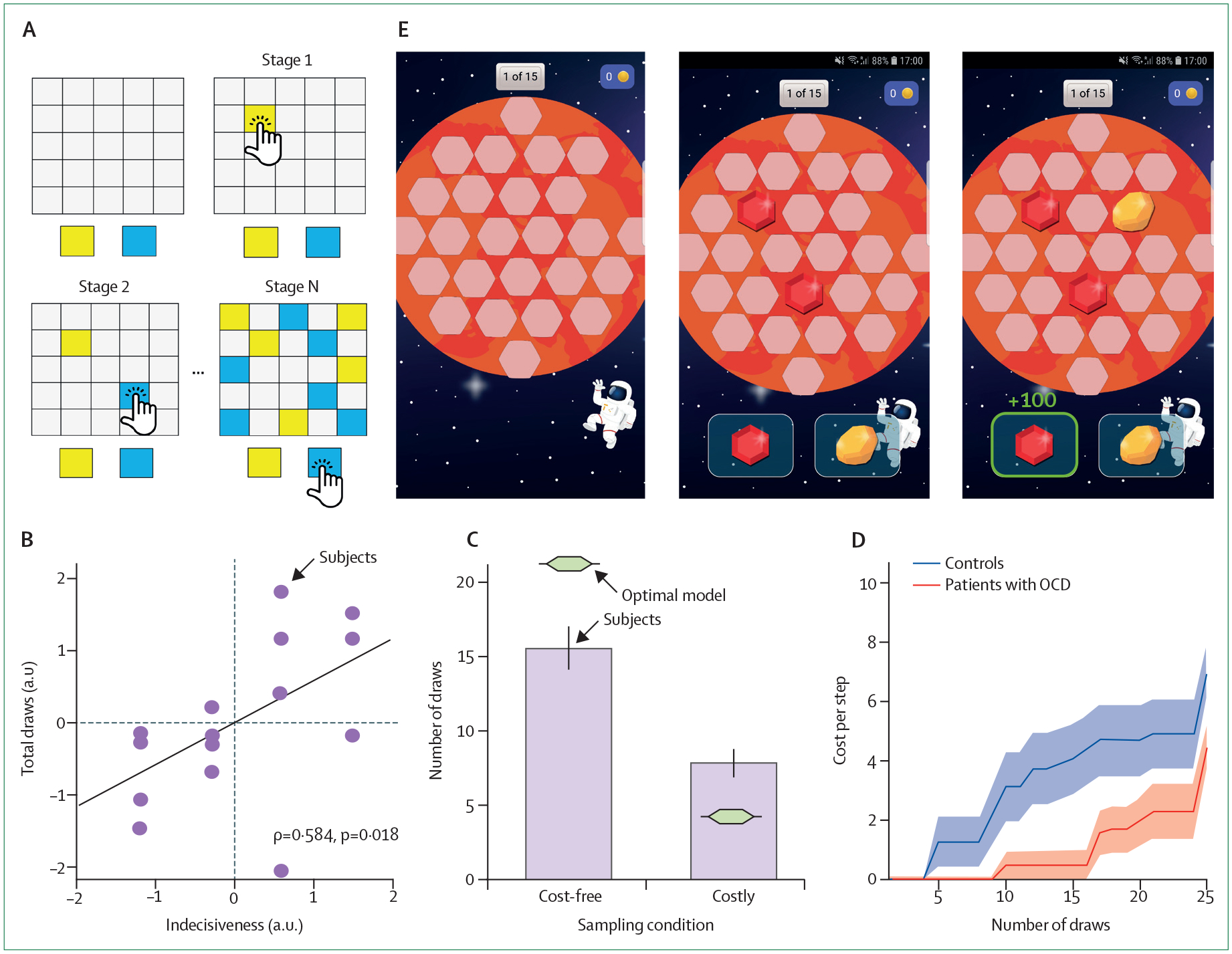
Computational modelling of indecisiveness (A) Laboratory information gathering task in which a participant is asked to determine which of the two colours is the more plentiful by drawing cards on the board. (B) This task-based measure of indecisiveness is linked to indecisiveness as assessed using traditional clinical interviews and showing ecological validity. (C) Computational modelling of drawing behaviour revealed that humans are suboptimal when making their decision, gathering too little information when it was cost-free, but gathering too much when information collection was costly. (D) Best fitting models showed that participants accumulate subjective costs that promote early decisions, and a bias in this accumulation process was driving the difference between participants with and without with obsessive compulsive disorder. (E) Gamification of this task allows the assessment of indecisiveness outside the laboratory in large samples of diverse backgrounds using smartphone apps, such as Brain Explorer. Parts B and D were reproduced from Hauser et al^48^ and were published under a creative commons attribution (CC BY). (E) from Brain Explorer app (www.brainexplorer.net). OCD=obsessive compulsive disorder.

Pervasive indecisiveness present in patients with obsessive-compulsive disorder^41–44^ is traditionally assessed using clinical interviews; by contrast patients with schizophrenia who show a jumping to conclusions.^45–47^ To objectively measure patient indecisiveness, we and others have used information gathering tasks (figure 3) to assess how much information participants accumulate before making a decision. Using Bayesian computational modelling, we can quantify how much they deviate from optimal behaviour^48^ and allow to closely capture participants’ behaviour. Because model parameters are well defined and functionally transparent, one can directly compare these model parameters and identify biased cognitive processes in developmental cohorts and patients.^48,49^ Moreover, by pairing modelling with causal brain-related interventions, such as pharmacological treatments, one can investigate the role of different brain and neurotransmitter systems in specific computational processes, such as indecisiveness.^50^

Although mechanism-driven models facilitate a better understanding of which neural or cognitive processes are impaired in patients these models are not yet used to predict psychiatric phenotypes (diagnoses and outcomes) in clinical practice. Most models are used to find differences between groups, rather than using these model parameters to estimate an individual’s psychiatric status. Studies suggest that mechanism-driven, model-derived parameters are better at predicting disease status or longer-term outcomes than standard neural, behavioural, or sometimes even clinical predictors^51,52^ (with balanced out-of-sample accuracies of up to 80%). However, how well these mechanism-driven models perform compared with mechanism-agnostic models, and how they can be supplemented with other data sources is yet to be determined.

### Mechanism-agnostic computational models

Since the advent of modern machine learning methods there has been considerable enthusiasm for their use, including deep learning, for precision psychiatry. Unlike mechanism-driven strategies, mechanism-agnostic models are usually complex with hundreds or thousands of free parameters. These models have achieved previously unseen performance in a wide range of tasks, from image classification to predicting protein structures.^53–55^

In mental health, mechanism-agnostic models are being used together with different forms of data, including clinical records, brain-based measures, and passively collected smartphone or social media data (panel 2). The aim of most of these studies is to predict mental health status, either a specific future psychiatric disorder, or a specific mental health syndrome, such as suicidality.^56^

#### Clinical data

With an ongoing digitalisation of health-care records across health-care systems, large clinical datasets for mental health are becoming available for interrogation. Although these datasets are sometimes limited in terms of data quality, organisation, and accessibility, as described by Koutsouleris and colleagues in Series paper 2,^11^ several studies have used mechanism-agnostic models with the primary aim of condensing and distilling information about mental health status and symptoms.^57^

In psychiatry, large amounts of clinical notes and medical records are difficult to condense because much of the relevant information is captured in the clinician’s notes, rather than in laboratory test indicators (eg, inflammation markers). Studies have successfully used natural language processing (NLP) algorithms, which allow the extraction of specific information from written text to help predicting outcomes, such as hospitalisation duration, readmission likelihood,^58,59^ and risk of suicide^60,61^ (with out-of-sample area under the receiver operating characteristic curve prediction from 0·58 to >0·80). However, these studies also make another key challenge apparent: what language features should these algorithms be trained on? Training NLP algorithms on specific language features relevant to psychiatry, such as research domain criteria-related content, might help improve these predictive models over standard semantic corpus labels.

It is relevant to note that mechanism-agnostic models are not confined to written notes. These approaches also hold great promise for more complex data, such as audio and video recordings from assessments and therapy sessions. These algorithms could assist clinicians by alerting them to subtle (emotional) reactions and other features that might go unnoticed.^62^

#### Complex research data

Scientific investigations of patients with psychiatric disorders often generate large data sets with many datapoints per participant. Neuroimaging (eg, MRI) data contain tens of thousands of datapoints per participant. This high dimensionality poses considerable challenges for analysing the data with traditional statistical approaches. Mechanism-agnostic models have been used mostly in two distinct approaches, either using data directly to classify and predict participants’ mental health, or using unsupervised (eg, clustering or factorisation) algorithms to create lower dimensional features, which can then be used for linkage with mental health status.

To predict current or future mental health status, many studies have used different variants of MRI data^10,63,64^ and deployed a wide range of machine learning models (with an out-of-sample predictive accuracy of usually >70%). Although these methods can discriminate between groups (eg, between patients and controls), newer studies have shown that these predictions improve significantly when integrating neuroimaging data with other data sources, such as clinician ratings, genetic data, and neuropsychological tests.^64^ This complementarity of neuroimaging data to other data sources also has implications for interpretability, because it allows a better understanding of the degree to which different sources are complementary, and how mechanism-driven features might shed light onto mechanism-agnostic features.

An alternative approach to analysing neuroimaging data is to use unsupervised models to generate low-dimensional brain organisation patterns, which can then be used to predict mental health status. Various methods have been used to generate such brain fingerprints, from clustering algorithms to canonical correlation analyses combining brain and behaviour to deep autoencoders.^65–70^ An advantage of these methods is that the intermediary brain fingerprints are often more interpretable and less noisy than when predicting mental health status directly from raw data, which can also help us to better understand the mechanisms underlying a specific status. For example, by using deep autoencoders of diffusion tensor imaging data, Chamberland and colleagues^66^ were not only able to predict various neurological and psychiatric disorders (area under the receiver operating characteristic curve from >0·6 to >0·8), but also generate anomaly metrics that allowed them to establish which fibre tracts were most relevant for each disorder.

#### Digital phenotyping

The use of digital data for predicting mental health has seen a substantial increase over the past few years. Because smartphones and social media are ubiquitous in our lives, they have become promising tools for collecting large amounts of data from participants capturing their dynamic real-world experiences;^70^ thus, smartphones are becoming ideal companions for data-hungry models. Many different types of measures can be extracted from digital data (panel 2). Generally, one distinguishes between passively collected or unobtrusive data, which do not require active responding by the participant, and active data collection, for which the participants are requested to engage (eg, mood self-reports). An advantage of passive data collection is that they only require minimal contributions from the participant, which greatly improves study compliance enabling efficient longitudinal data collection.^13^

Mental health has been linked to various types of passively collected data, including geolocation,^71,72^ sleep disturbance data,^58,73^ and smartphone usage patterns.^15,74^ Although the passive data collected using smartphones might not be as informative as in-depth clinical measurements, the minimally invasive nature over longer time periods might lead these data to be considered to be as valuable as more costly data acquisition methods, especially when combined across multiple data sources. Of particular interest are data from social media, such as usage patterns or content of messages. These data have been used to predict mental health status and outcomes,^56^ as well as the likelihood of upcoming readmission to hospital. The wide range of predictive accuracy in these studies is likely due to different data sets, data features, and time horizons.^75^

The promise of using digital data is substantial and evidenced by a surge in research papers.^56^ This trend can be observed in many start-ups entering this field, and technology giants, such as Facebook, already using similar models for suicide prevention on their platforms.

## Building useful models

### Barriers for models to become useful

Both mechanism-driven and mechanism-agnostic models have shown their potential for psychiatry. However, unlike other fields (eg, judicial system),^76^ few models have found their way into clinical practice.^77^ Of note, mechanism-driven and mechanism-agnostic models seem to have distinct implementational constraints and difficulties.

For mechanism-driven models, a key challenge is their predictive performance. Traditionally, mechanism-driven models are developed and optimised to capture behaviour or neural responses. Because these models are not optimised to predict mental health status, ideal therapeutic response, or long-term outcomes, these parameters often display a more restricted predictive power than models optimised to predict mental health-related phenotypes. Attempts to overcome this weakness use generative embedding strategies, which use mechanism-driven algorithms as a dimensionality reduction step before the subsequent generation of optimally predictive mechanism-agnostic models.^78^ Another limitation of mechanism-driven models is that many rely on complex data collection, which substantially restricts their use outside of academic settings.

For mechanism-agnostic models, the key challenge is understanding how these models operate and what they predict. Their complexity renders them opaque,^79^ but improving our understanding of them is crucial for three reasons: (1) only through understanding mechanism-agnostic models will we be able to establish which input variables are relevant and which could be removed, which is challenging in complex and non-linear mechanism-agnostic models; (2) understanding enables us to detect biases and faults of the model that arise through biased training sets;^11^ (3) predictions from unexplainable models can pose a substantial challenge when used in clinics because the uptake of model predictions strongly depends on clinical staff understanding and trusting them. We propose to use three strategies that could help alleviate these limitations.

#### Translation: from the laboratory and into the real world

Many mechanistic assessments, such as computational psychiatry and neuroimaging tasks,^80^ have only been evaluated in small samples of highly selected participants, and little is known about their potential for predicting mental health status in real-world clinical cohorts. Therefore, we need to examine the use of mechanistic assessments outside of overly selective laboratory samples in large, epidemiologically sampled populations.^11^ This is crucial because these assessments still rely heavily on the experimenters’ instructions. For any assessment that should be applied to clinical practice, assessments that are robust to experimenter biases are required (panel 3). In addition, long assessments using expensive neuroimaging methods are unlikely to become clinically viable; this means that proxies that substitute these measures in clinical settings require development.

A move towards online-based task assessments over the last decade constitutes a first step towards clinically usable data assessment tools.^88,89^ Using online worker platforms, researchers have developed methods for instructing complex tasks that are entirely digital,^2,39^ showing similar behavioural patterns as observed in the laboratory.^86^ However, paid participants on such platforms are often professional experiment participants, and might not reflect the population that these tests will be used in.

Consequently, studies have now entirely departed from traditional participant pools towards more population-reflective, crowd-sourced data collection. The use of gamified smartphone applications (eg, Brain Explorer, Great Brain Experiment [UCL, London], and Neureka [Trinity College, Dublin]) has proven to be promising.^86,90–92^ By recruiting participants worldwide and from diverse demographic backgrounds, such big data approaches open promising new avenues for collecting data that are more representative of the reality encountered in clinics.

Although gamified approaches are unable to replace neuroimaging markers directly, they can help to inexpensively approximate potential mechanisms. By using similar tasks used in neuroimaging scanners, we can use computational models to infer the probable neural mechanisms relevant for imbalanced processing. Moreover, by using pharmacological manipulations, we can obtain relevant information about possible neurotransmitter involvement that can be helpful for pharmacological treatment predictions.^43,93^

A key advantage of mobile assessment platforms is that they are more amenable for repeated and triggered assessments. They can be combined with self-reports collected as ecological momentary assessments. In addition, bringing assessments together with passive data collection or physiological data, such as pupillometry,^94^ might provide additional crucial information.

#### Explanation: from black to grey boxes

The inability to understand many mechanism-agnostic models not only challenges their usability, but also threatens their uptake in clinics and might become a regulatory requirement. Over the past few years, various techniques, predominantly in image classification, have been developed trying to explain black box models (eg, deep dreaming^95^ and attention maps^96^). However, these explanations are not undisputed because they only provide an approximation to the true model. This means that they are unable to fully capture the model and could provide false explanations for a considerable number of cases.^79^

Complementarily, an important new trend in machine learning is the use of causal models that allow advancement beyond simple correlational effects. This is particularly relevant in psychiatry to identify factors that are causal for mental health and not simply coincidental. Although there are several different forms of models that allow the assessment of causality,^97,98^ methods for more complex mechanism-agnostic models are only slowly emerging.^99,100^ Therefore, it is important to build mechanism-agnostic models that are transparent by selecting interpretable algorithms by design (eg, XGBoost).

Another method to increase interpretability is to use dimensionality reduction approaches before using these lower dimensional features for prediction. this modularisation is useful to assess the performance of each compartment independently and exploit the relatively low dimensionality of the final prediction model to establish better understandability. An example of such an approach is the prediction of psychosis onset, in which a combination of separately aggregated clinical, neuroimaging, and neuropsychological predictors have revealed partly additive and explainable effects.^10^ Therefore, it is important to carefully consider the complexity of a model and to balance interpretability and complexity in accordance with the demand.

#### Combination: bringing together different sources and models

**Figure 4:**
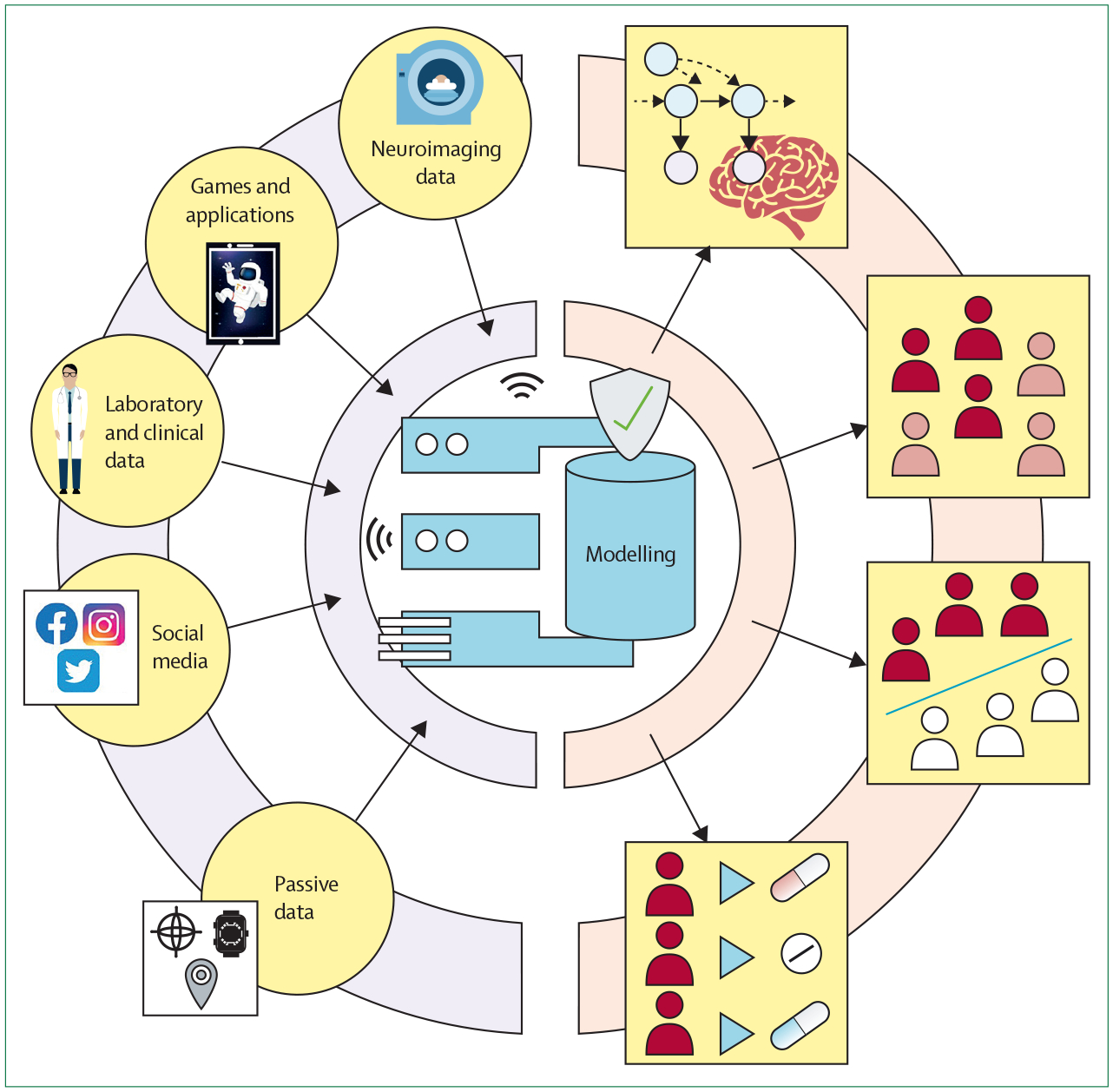
Bringing data sources together to improve modelling in psychiatry Although most research has focused on single data sources for their models, bringing complementary data sources together can help improve model performance. Therefore, mechanism-driven model indicators can help with the interpretability of black box models. Substituting complex in-laboratory data sources with more readily available proxies, such as smartphone-based games, can help bring research-led findings into a real-world setting. These extended strategies might help build clinically useful models.

Thus far, the computational modelling in psychiatry mainly consists of many scattered, independent approaches to explain mental health, but these different promising attempts have not yet been brought together. Building clinically useful models will require us to overcoming these fragmented aspirations to pursuing the integration of different data sources following modelling strategies that maximise complementarity and interpretability (figure 4). For example, for treatment prediction and stratification, a series of person-specific and disorder-specific factors that predict success in treatment are known. Task-derived mechanistic models^101^ and digital markers^56,101–103^ could complement such data and improve performance.

When approaching data integration, it is crucial to be aware of the complementarity of the data. Data sources that capture entirely distinct data types (eg, computational tasks) are likely to be non-overlapping and thus add meaningful new dimensions that can help elucidate mental health heterogeneity. Therefore, by combining these different data sources and models, we might be able to more comprehensively parametrise a person’s mental health.

Focus should be directed towards mechanism-driven models and data sources that extract meaningful features of rich data; by bringing these data sources together in shallower and interpretable mechanism-agnostic models, we will be able to identify the role of each of these condensed features. Such approaches also allow us to assess which data sources contribute to prediction the most, and which can be eliminated without losing predictive power. The first attempts for fusing different data and modelling modalities show promise,^64,96^ but their clinical usefulness is yet to be determined. Moreover, by bringing together mechanism-driven and mechanism-agnostic models, we can detect shortcomings of our mechanism-driven models and improve our mechanistic understanding.^104^

## Conclusion

A wealth of computational approaches to psychiatry make navigating this complex, rapidly evolving space challenging and understanding the uniqueness versus the relatedness of these models more difficult. A stricter standardisation of modelling strategies and enforcement of comparability is needed to achieve a transparent landscape of computational modelling in psychiatry. In this Series paper, we show how to dissociate these models based on their purpose. Moreover, we have highlighted the importance of bringing these disparate models and data sources together to increase both prediction and interpretability. In particular, the combination of mechanism-driven and mechanism-agnostic models hold great promise to derive biologically informed and transparent prediction models, which could help to develop novel treatments and interventions.

## Panel 1: Glossary

### Bias-variance trade-off

A conflict between two types of errors that must be minimised when developing a computational model. Bias error arises due to underfitting and the model not capturing the relevant associations between features and output labels. Variance error mostly arises if the model is overfitting the training set and interprets random noise as meaningful variation.

### Computational psychiatry

A field of research that seeks to characterise mental dysfunction in terms of aberrant computations over multiple scales.

### Cross-validation

A procedure to split data into train and validation sets to provide unbiased estimates of model performance. The model is fitted on the training data and evaluated on the validation data that were not used to fit the model. This procedure is k-times repeated until all the data has been used in the test data once (eg, k=5-folds) to evaluate model robustness and prevent overfitting.

### Deep autoencoders

Class of deep learning algorithms that have found great use for unsupervised learning problems. Using two symmetrical deep networks, the autoencoders are optimised to produce output data that resemble the input data via a compressed representation of the latter.

### Deep learning, deep neural networks

A branch of machine learning that uses multilayer artificial neural networks to derive model predictions. Stacking several layers of artificial neurons on top of each other allows fitting of highly dimensional data with complex non-linear relationships, but it comes at the cost of increased model complexity (can reach several million free parameters) and problems with interpretability (black box models).

### Dimensionality

Dimensionality of the data is determined by the number of input features. Highly dimensional data are rich in information and potentially more robust against noise, but they often require special models that can account for the redundancy and high covariance between features (eg, regularised models).

### Diffusion tensor imaging

An MRI technique used to estimate the white matter (axonal) organisation of the brain.

### Ecological momentary assessment

Methods of repeated sampling of an individual’s behaviour and experiences in real-time and in natural environments.

### Effect size

A quantity that measures the strength of the dependency between dependent (features) and independent (labels) variables.

### Functional MRI

An MRI technique that indirectly measures brain activity by registering differences in blood oxygen level. This is a common method to understand the functional neural organisation of cognition and behaviour.

### Gamification

Describes the approach of making cognitive (and other) tasks more game-like using principles and design elements successfully used in electronic games with the goal of making them more entertaining and, therefore, increase user engagement.

### Model features

Data or an aggregated substrate thereof is used to train a computational model to predict a label. Features could be continuous (numerical, such as height or weight of an individual), categorial (eg, gender), or more complex, such as text strings, graphs, or multidimensional syntactic features.

### Model fitting

A process of finding model parameters so that the model’s predictions maximally resemble the data (parameter optimisation). Model fitting can be implemented through different optimisation methods.

### Model labels

Outcome variables that the model tries to predict. Similarly, to model features, model labels could be numeric (continuous, such as the duration of hospitalisation), discrete (eg, whether an individual will develop a psychiatric disorder or not), or more complex entities (eg, the next word in a sentence).

### Model selection

An important step in finding good computational models for mental health is to establish the best model among a pool of different possible models for the data given. Traditionally, selection considers the model fit and the model complexity, to avoid overfitting and underfitting. However, in this Series paper we discussed other aspects of model selection, such as mechanism-agnostic versus mechanism-driven models or interpretability of models that are less easily quantifiable. Therefore, it is crucial for researchers to have a clear understanding of the advantages and disadvantages of the models chosen, and to use the adequate selection criteria.

### Natural language processing

A branch of machine learning focused on the algorithms that process, predict, and generate natural language and speech.

### Occam’s razor

A principle of constructing explanations formulated by scholastic philosopher William Ockham in the 13th century which states that “*pluralitas non est ponenda sine necessitate*”, translated to “plurality should not be posited without necessity.” This principle favours a simple theory or model over more complex one if the former can explain the phenomenon.

### Overfitting

A situation when the model explains the data too well because of poor model fitting procedure. Overfitting describes when the model takes the negligible deviations in the data into account and is unlikely to perform well with unseen data.

### Parameter optimisation

A set of procedures of finding a set of parameter values in the model that will maximise the model’s objective function (eg, likelihood).

### Precision psychiatry

An approach for the treatment and prevention of psychiatric disorders that considers individual variability in genes, biology, cognition, environment, and lifestyle.

### Recurrent neural networks

A term used for two separate types of models: (1) a type of artificial neural networks that allows for information to be retained over time enabling memory in these networks and (2) network models with multiple artificial neurons that are connected to each other.

### Regularisation

A set of constraints that are imposed on model parameters (eg, weights or coefficients) to prevent them from taking large values. These techniques stop the model from putting too much weight on some of the features, reduce model complexity, and help prevent overfitting.

### Reinforcement learning

A domain of artificial intelligence that focuses on building intelligent agents who learn by trial and error to take actions that maximise their cumulative future reward.

### Reliability

Quantifies how consistently a method or a model measures a phenomenon of interest (see panel 3: challenges).

### Spiking neural networks

A type of artificial neural networks that incorporate spiking properties of natural neurons.

### Support-vector machines

A type of machine learning supervised algorithms that are used for classification and predictive modelling. Support-vector machines construct linear and non-linear hyperplanes, which allow for separate data points to be put into different classes.

### Validity

Quantifies how accurately a method or a model measures a phenomenon of interest.

## Panel 2: Data sources

Computational models in psychiatry have used a wide variety of different data sources, and they substantially differ in their advantages and disadvantages.

### Clinical data

Data collected in the context of mental health care can range from hospitalisation duration to detailed notes on the patient from clinical staff. However, privacy concerns and missing data infrastructures make it challenging to harvest such data for modelling purposes.^11,12^

### Laboratory-based data

Data collected in controlled environments for scientific studies. These often entail behavioural and biology-derived data. Due to the well controlled settings and often selective participant recruitment, noise in the data is reduced to yield maximal effect sizes. However, sample sizes due to expensive data collection methods are often restricted and translation to clinics is challenging as models are not prepared for the increased heterogeneity and noise in real-world clinical samples.

### Digital data

Digital data can be roughly divided into passive and active data and includes any data that was collected from the participant using digital devices.^13^ Most commonly used data stems from mobile phones and social media.

- Active data requires the participant to interact with a request from the experimenters. Most common are probes that assess one’s self-reported mood and experiences. A second promising avenue are game-like cognitive assessments with smartphones. These short games help overcome the limitations of laboratory-based studies and to collect large samples.^14^ Such approaches also allow for repeated longitudinal assessments and context-specific assessments.
- Passive data does not require the participant to respond to the study, which has the advantage that participants are less likely to drop out and such data collection is well suited for longitudinal studies.^13^ Data ranges from social media activity and communication patterns to sensor data from smartphones and wearable devices. Social media activity and geolocation data has been particularly popular in mental health research,^13,15,16–18^ but other data sources, such as light sensors, voice recordings, accelerometers, and physiological recordings, also hold promise. Bringing together passive and active data sources, for example by collecting eye gazing data during game play,^19^ could yield new insights in future studies.

## Panel 3: Challenges for computational models

Despite the plethora of computational models in psychiatry, they are all built on the same computational pillars and face similar challenges.

### Noisy data

Noise in the data affects model quality and reliability and can add bias. Measurement noise can arise from participant inconsistencies (particularly in poorly controlled data collection environments),^81^ imprecise data collection (eg, MRI artifacts), or insensitive task measures.

### Missing and sparse data

Missing data is one of the main concerns for model building and often requires additional statistical preprocessing and corrections, especially in longitudinal studies.^82^ Sparse data (eg, imbalanced samples) can lead to substantial biases even in large data sets and especially for minority populations.^14^

### Validation

When using computational models, it is crucial that the model’s performance is validated against an independent test dataset (out-of-sample prediction, cross-validation). If such an approach is not used (ie, within-sample prediction), then the accuracy might be inflated and the results are prone for overfitting. Validation is crucial in contexts in which, besides the model parameters, hyperparameters are optimised, which requires a careful delineation of datasets.

### Small sample size and reproducibility

Laboratory-based studies tend to include smaller and biased sample sizes, which could lead to non-reproducible effects and low statistical power.^83–85^ A solution to this can be online or smartphone-based data collection,^86^ which is particularly promising for assessing game-like computational tasks.

### Reliability and validity

For computational models to produce generalisable and replicable results, it is important that the assessments produce reliable results. Unfortunately, little is known about psychometric properties of computational models and their data sources. Studies have assessed the reliability of tasks and introduced methods and task-related measures to improve reliability.^87^ Similarly, a low reliability of psychiatric diagnoses renders prediction more difficult.^8^

### Temporal dynamics

Many data sources (mental health symptoms, brain activity, cognitive variables, and social media) show fluctuations and oscillations on different time scales (from seconds to years). These temporal dynamics might be disorder relevant or entirely independent. Because these dynamics cannot be detected when using cross-sectional or temporally sparse assessments, it is important to use repeated longitudinal assessments to assess and exploit these dynamics for modelling mental health.^19^

### Generalisability

Generalisability describes the ability to use models beyond the data that were used to develop the original model (ie, predicting the labels correctly in new data). This is crucial for the clinical success of modelling efforts, but also a key challenge especially if the data samples differ substantially from clinical reality.

## Search strategy and selection criteria

Ideas and content of this Series paper were developed through a series of discussions evolving between the authors and taking place between May and December, 2021. The wide angle of perspectives on the development and integration of AI tools in future mental health care led to the formulation of two primary foci on modelling and implementation challenges of AI in mental health care. The two primary foci were discussed in-depth, respective content ideas were grouped into sections, and keywords for literature search on PubMed and Google Scholar were formulated for each section. For this Series paper the search terms to gather the published material associated with mechanism-driven models were [“computational psychiatry”, (“computational model” OR “Neural network” OR “Reinforcement Learning” OR “Bayesian model”) AND (“Psychiatry” OR “Mental Health” OR “Psychiatric”)], for mechanism-agnostic models the search terms were [(“machine learning” OR “Computational Model”) AND (“Psychiatry” OR “Mental Health” OR “Psychiatric”)], and for models using digital data the search terms were [(“social media” OR “smartphone”) AND (“machine learning” OR “Computational”) AND (“Psychiatry” OR “Mental Health” OR “Psychiatric”)]. In addition, relevant references from the obtained papers were also considered. Obtained papers (published after 2010) were non-systematically selected based on the representativeness of the work for the given section topics and based on the quality of research. The selection of references was discussed and supplemented as required to substantiate the authors’ viewpoints from different perspectives. The focus was on papers published in English, but papers in languages the authors spoke were also considered.
